# The Psychological Impacts of COVID-19 Home Confinement and Physical Activity: A Structural Equation Model Analysis

**DOI:** 10.3389/fpsyg.2020.614770

**Published:** 2021-01-15

**Authors:** Xuehui Sang, Rashid Menhas, Zulkaif Ahmed Saqib, Sajid Mahmood, Yu Weng, Sumaira Khurshid, Waseem Iqbal, Babar Shahzad

**Affiliations:** ^1^School of Physical Education and Sports, Soochow University, Suzhou, China; ^2^Postdoctoral Researcher, Research Center of Sports Social Sciences, School of Physical Education and Sports, Soochow University, Suzhou, China; ^3^College of Management, Shenzhen University, Shenzhen, China; ^4^Department of Zoology, Hazara University, Mansehra, Pakistan; ^5^School of Education and Science, Neijiang Normal University, Neijiang, China; ^6^School of Energy, Soochow University, Suzhou, China

**Keywords:** COVID-19, psychological impact, lockdown, physical activity, China

## Abstract

**Background:**

COVID-19 break out has created panic and fear in society. A strict kind of lockdown was imposed in Wuhan, Hubei province of China. During home confinement due to lockdown, people face multidimensional issues. The present study explored the psychological impacts of COVID-19 home confinement during the lockdown period and Wuhan’s residents’ attitude toward physical activity.

**Methods:**

A cross-sectional online survey was conducted to collect the primary data according to the study objectives. The population was Wuhan residents (+ 18 years) who were in home confinement. A total of 2280 participant’s reply to the online questionnaire survey and collected data after quantifying the data, about 2200 (96.49%); about (49.8%) female, about (49.4%) male, others about (0.2%), and about (0.6%) were not disclosed their gender participants responses were used for analysis. The collected data were analyzed through appropriate statistical techniques.

**Results:**

According to results, H1 is supported with β = −40.793, *t* = 57.835, *p* = 0.000, which claimed a negative association between COVID-19 lockdown policy and behavior and attitude. Results for H2 reveals that the COVID-19 lockdown policy have negative influence on emotional control with β = −0.769, *t* = 46.766, *p* = 0.000 and it is supported. H3 documented a significant positive relationship between COVID-19 lockdown policy and lockdown period psychological impact, which means lockdown policy, is the main reason to increase the lockdown psychological impact. Further, COVID-19 lockdown policy have negative influence on physical activity (H4) and self-belief (H5) with β = −0.657, *t* = 32.766, *p* = 0.000 and β = −0.620, *t* = −6.766, *p* = 0.000 respectively. H6 stated that there is a positive impact of behavior and attitude toward physical activity. The results for H6, behavior, and attitude affecting the physical activity with β = 0.401, *t* = 10, *p* = 0.000, which is supported.

**Conclusion:**

COVID-19 home confinement created various psychological impacts, negatively affecting the emotional state due to depression and anxiety. Physical activity is the best strategy to manage human nature’s psychological issues, and people’s attitudes were positive toward physical activity during home confinement. However, the lockdown policy also affects physical activity participation negatively, and a sedentary lifestyle prevailed during home confinement.

## Introduction

A novel beta coronavirus (COVID-19) emerged in Wuhan, Hubei province of China, which caused a worldwide public health emergency. It was declared a pandemic by the World Health Organization (WHO) due to its severity, which caused panic and anxiety (Pradhan et al., 2020). According to the WHO, the COVID-19 has spread across the world, and currently, 213 countries are facing different measures imposed by their governments to contain the COVID-19. On January 23, 2020, the Chinese Government, in the face of such a severe epidemic, implemented a variety of steps to prevent and control viral transmissions, such as the lock-out of entire cities, travel warning regulation, and home medical observations (Anna, 2020). Limitations on travel and outdoor leisure will eventually disrupt Chinese people’s daily routine and lifestyle. People would also be less physically active, more sedentary, and more depressed, which could pose substantial risks to safety and wellbeing (Chen et al., 2020).

The lockdown policy implementation across China created many socio-psychological issues for the Chinese people in every life segment. During the lockdown period, people were remained confine at home to contain the pathogen. Home confinement has a long-lasting negative psychological impact on mental health and wellbeing. Chinese people faced anger, boredom, and loneliness during home confinement, increasing psychological issues such as depression, stress, and anxiety (Duan and Zhu, 2020). Different kinds of outbreaks, such as MERS, Ebola, and swine flu, caused multiple psychological impacts (Rubin et al., 2010; Al Najjar et al., 2016). Furthermore, Wang and Zhao (2020) reported higher anxiety among graduate students during the COVID-19 outbreak, which further created psychological impacts on their academic life. Limitations on travel and outdoor leisure will eventually disrupt Chinese people’s daily routine and lifestyle. People would also be less physically active, more sedentary, and more depressed, which could pose substantial risks to safety and wellbeing (Chen et al., 2020). Quarantine brought multiple psychological impacts on people’s lives, such as fear of infection, financial loss, boredom, and frustration (Brooks et al., 2020).

Home confinement has negative socio-psychological impacts on mental and physical health. Long term isolation creates negative emotions, impaired cognition, and stress (Hawkley and Capitanio, 2015). In the context of the COVID-19 pandemic negative impacts, a study conducted in Spain reported that adverse psychological effects have occurred on Spanish people, and women and young people suffered most (Rodríguez-Rey et al., 2020). Sleep quality is also negatively impacted by COVID-19. A study conducted by Xiao et al. (2020) reported a negative relationship between quality of sleep and psychological impacts. The WHO has recommended various protective measures to stop the transmission of COVID-19, and physical activity while staying at home is also one of the protective measures. Social isolation is essential as the global response to the pandemic of COVID-19 continues. In everyday tasks, the physical distance from others is conserved. The criteria for participation in physical activity have drastically changed. COVID-19 should have had a huge social impact as opposed to years of public safety lobbying. Many individuals rediscover the pleasure and happiness of exercise activities (Tison et al., 2020).

Previous studies regarding different outbreaks reported that the psychological impact of confinement differs from immediate effects such as impulsivity, frustration, uncertainty, dissatisfaction, isolation, depression, anxiety, denial, insomnia, the desperation to extremes of consequences, like suicide, fear of contracting, and transmitting the infection to family members (Robertsons et al., 2004). Compared with their sedentary peers, physically active people are less at risk for certain disorders, including cardiovascular diseases (Murtagh et al., 2010), metabolic disorders (Hamilton et al., 2014), and depressive symptoms. It is generally accepted that insufficient physical activity is a significant risk factor for cardiovascular diseases, elevated blood pressure, asthma, and breast and colon cancer. Physical inactivity is the leading cause of mortality across the world. The extended homestay can lead to increased sedentary behavior, such as spending too much time sitting down, reclining, lying down for screening habits, and often reducing physical activity. There is a clear health argument for continued physical activity in the home to remain healthy and preserve immune system function in the current precarious climate. Even though the virus pandemic is an immediate public health concern as soon as possible, there are few recommendations for public health as to what people should or can do to preserve their regular practice or physical activity habits. It is possible that an unintended negative effect can occur when remaining at home since efforts to prevent the transmission of the virus from human to human may lead to a reduction in physical activity (Owen et al., 2010; Chen et al., 2020). With the COVID-19 outbreak, some researchers have shown a good health case for maintaining physical activity at home to retain a healthy immune system function (Martin et al., 2009).

High-intensity physical activity results in the form of an immunosuppressant (Wetmore and Ulrich, 2006). Recent studies have suggested that daily physical exercise boosts the immune system and enhances resilience to infections (Gleeson, 2007; Nieman and Wentz, 2019). Besides, it is a physical health concern and a psychological disorder, given the context of social isolation and the perception of alienation. In older adults, even psychological distress symptoms were expected and may lead to psychiatric problems and a higher risk of psychological distress (Calati et al., 2019; Santinis et al., 2020). In the context of psychological wellbeing, physical activity and good quality of sleep have an association. Cross-sectional studies show that those adolescents who are physically active have good quality sleep than physically inactive adolescents (Park, 2014). In neurological disorder is based upon the central and peripheral nervous system diseases. These diseases are categorized as Alzheimer’s, epilepsy, dementia, migraine, brain tumors, neuro-infections, and traumatic disorders. Physical activity also has a good impact on neurological functions. During physical activity, our brain mechanism also positively affects the molecular and cellular levels, improving brain health (Neufer et al., 2015). Research studies on aging populations show that cognitive decline can be protected through physical endurance activity (Boucard et al., 2012). Physical activity is a catalyst to improve the central nervous system, such as traumatic brain injuries and stroke. In the traditional context, physical activity enhances the brain’s functions through nerve and muscle enhancement (Stroud et al., 2009).

**FIGURE 1:**
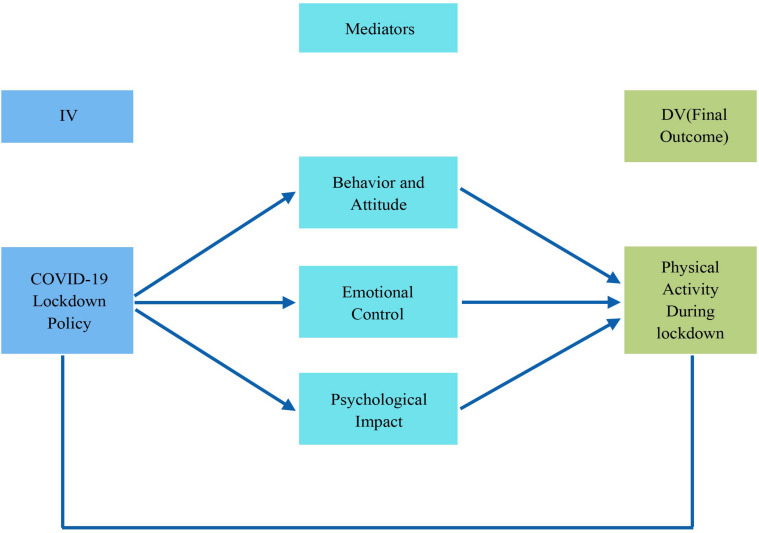
The relationship between different variables of the study.

The present study was designed to explore the psychological impacts of Wuhan residents’ COVID-19 home confinement and physical activity participation level during the lockdown period. Due to the various travel restrictions, outdoor physical activity venues such as playgrounds, dancing squares, and gymnasium were closed. Physical activity levels among the Chinese population but at the global level decreased due to the COVID-19 pandemic. Different exercises with numerous safe, convenient, and rapidly implementable technologies are perfect for preventing and remaining active in the airborne coronavirus. Exercise at home includes walking around the house and store, carrying things, scaling stairs, spinning, sitting and sitting on a chair and from the floor, squatting chairs, sit-ups, and push-ups. Also, as they require little facilities, space and are feasible in all circumstances, classical Tai Ji Quan, Qigong exercises, and yoga should be considered. Sport is a powerful Chinese society component for encouraging a healthy living style (Dai and Menhas, 2020). Other feasible ways of preserving physical function and psychiatric wellbeing during this crucial time include the use of e-Health and exercise videos that encourage and provide physical activity through the internet, mobile technology, and TV. Homestay is a vital measure for the defense that can restrict the spread of pathogens. However, prolonged house stays can lead to inactivity and trigger anxiety and depression, leading to a sedentary lifestyle that can lead to chronic illness. Maintaining regular physical activity and exercising in a clean home environment is essential during the coronavirus epidemic for a healthy lifestyle. The present study explored the psychological impacts of COVID-19 home confinement during the lockdown period and its implications on Wuhan’s residents’ attitude toward physical activity. The relationship between different variables of the study has been shown in Figure 1.

**Hypothesis’s of the Study**

H1: COVID-19 lockdown policy (CLP) negatively influences the behavior and attitude (BA)
H2: There is a negative association between COVID-19 lockdown policy (CLP) and emotional control (EC)
H3: COVID-19 lockdown policy (CLP) positively influences the lockdown period psychological impact (LPPI)
H4: COVID-19 lockdown policy (CLP) has a negative influence on physical activity (PA)
H5: COVID-19 lockdown policy (CLP) has a negative influence on self-belief (SB)
H6: There is a positive effect of behavior and attitude (BA) on physical activity (PA) during COVID-19
H7: Physical activity (PA) positively influence through emotional control (EC)
H8: Physical activity (PA) positively influence through self-belief (SB)
H9: Physical activity (PA) positively influence through lockdown period psychological impact (LPPI)

## Materials and Methods

### Study Locale

**FIGURE 2:**
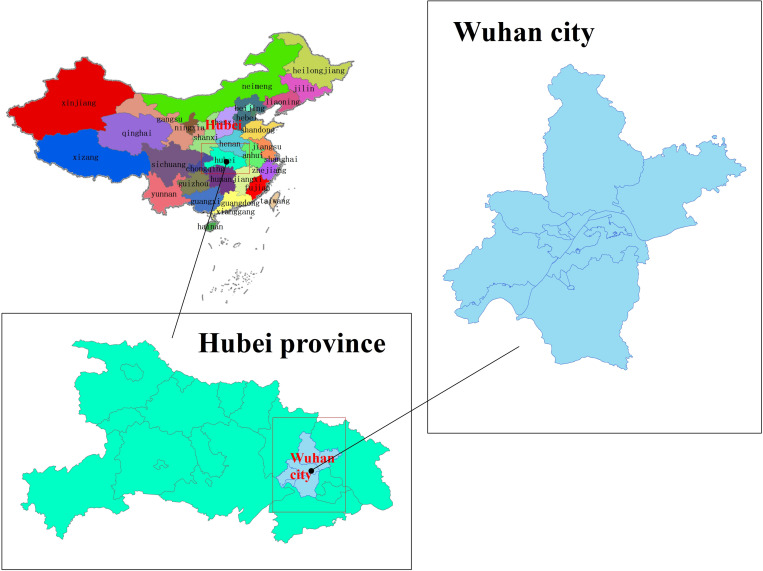
Study population.

The present study was conducted in Wuhan city, shows in Figure 2 below, the capital of Hubei province China. The research followed the principles of the Helsinki World Medical Declaration, and the ethics committee of Soochow University, Suzhou, approved the study.

### Sampling

The population was Wuhan residents (+18 years) who were in home confinement. A convenience sampling technique was used to collect the data through an online survey. A total of 2280 participant’s respond to the online questionnaire survey, and after quantifying the data, about 2200 (96.49%); about (49.8%) female, about (49.4%) male, others about (0.2%), and about (0.6%) were not disclosed their gender participants responses were used for analysis. The responses of the 80 participants were excluded because of missing data. The final sample for this study included 2200 surveys.

### Data Collection and Procedure

The survey method was used to collect the data, and the survey was developed after review the psychosocial impacts of previous outbreaks of influenza and SARS (Rubin et al., 2010). A cross-sectional online survey was conducted to collect the primary data according to the study objectives. All the survey participants were informed about the purpose of the study and then received their consent. The collected data were proceeding anonymously, and the researchers performed a quality check. The anonymity of the survey participants and collected information privacy were assured. During the informed consent, it was confirmed that all data were used only for research purposes.

### Measures

A cross-sectional online survey was conducted from February 20 to March 20, 2020, during Wuhan’s lockdown. The questionnaire was based on both open-ended and close-ended questions regarding participants’ socioeconomic information, the psychological impact of COVID-19, response to the COVID-19 psychological impact, and participation in physical activity during home confinement.

#### Demographic Characteristics

Considering previous studies (López-Sánchez et al., 2020), the study included gender, age, education, marital status, religious belief, ethnicity, and employment status during COVID-19 as demographic factors. All variables were self-reported and categorized as gender (male, female, others, prefer not to answer), education (less than a high school degree, associate degree, bachelor’s degree, graduate degree, others), marital status (Single; never married, married, or in a domestic partnership, widowed, divorced, separated married), religious belief (yes, no), ethnicity (Han ethnicity, national minority), and employment status (employed full time; including self-employed or homemaker, employed part-time; including self-employed or homemaker, unemployed, student, retired, unable to work).

#### The Psychological Impact of COVID-19

National lockdowns and home-confinement policies introduced in most COVID-19-hit countries after China prevent further spread of pathogens. A broad fraction of the global population is primarily limited to their homes (Rubin and Wessely, 2020). The lockdown period psychological impact (LPPI) was assessed by using Likert scale questions about feelings or thoughts during lockdown, behavior, and attitude (BA), self-belief (SB), and emotional control (EC).

#### Physical Activity

Physical activity is a catalyst for wellbeing and good health. Human biology requires a particular degree of physical activity to sustain good health (Leonard, 2010). Participation in physical activity was affected during lockdown due to the closure of parks, gyms, and public spaces. The survey participants participation in the physical activity was assessed through the questions (have you exercised, how many days in a week did you do vigorous physical activities, where did you do your physical activities most of the time during the Lockdown period, and physical activity levels during the lockdown period).

### Statistical Analysis

The collected data were analyzed using Statistical Package for Social Sciences (SPSS) version 23.0 (SPSS Inc., Chicago, IL, United States). The statistical analysis was divided into three parts, univariate, bivariate, and multivariate, to analyze the psychological impact of COVID-19 home confinement and physical activity to achieve the study objectives. Under the multivariate analysis, the structural equation model was used to assess the association between different variables regarding the psychological impact of COVID-19 home confinement, cope with psychological stress due to COVID-19, and physical activity participation COVID-19 home confinement. Moreover, the study employed a structural equation modeling technique (SEM) to examine various variables’ relationships. The PLS-SEM research design is a stable, versatile, and advanced tool for creating a significant statistical model, and the PLS-SEM role helps achieve the intended goal (Abbas et al., 2019). The structural equation modeling SEM technique was applied through the smartPLS 3.3.0 package. The structural equation modeling (SEM) also enables the study of linear associations between manifest variables and latent constructs (Aman et al., 2019). The structural equation modeling (SEM) for this study is characterized by six observed variables, as mentioned in Figure 1. The COVID-19 lockdown policy is used as independent variables, and mediators are behavior and attitude, emotional control, psychological impact, self-belief. In contrast, only physical activity during the lockdown period as dependent variables is used.

## Results

### Descriptive Analysis

#### Demographic Characteristics of the Survey Participants

A total of 2200 survey participants completed the online survey, and Table 1 shows the demographic characteristics of the target population. According to the descriptive statistics of the sample are depicted, the majority of the survey participants, about (49.8%), were female while about (49.4%) were male, others were about (0.2%), and about (0.6%) were not disclosed their gender. Age statistics show that the majority of the participants about (37.7%) belongs to the age group of 18–24, about (18.8%) falls in the age group of 45–54, about (17.7%) in the age group of 35–44, and about (10.8%) belong to the 65 + years, while only about (8.8%) participants in the age group of 25–34. Educational information of the survey participants shows that majority of the participants, about (27.0%) are holding graduate-level education, about (26.4%) have a bachelor degree, about (22.3%) have less than a high school degree, and about (14.7%) have an associate degree, while only about (9.5%) about other kinds of education such vocational and technical skills education. In the context of marital status, the majority of the participants about (50.3%) are married or in a domestic partnership, about (4.9%) are widowed, and about (2.6%) divorced. In comparison, only about (0.5%) are separated from their life partners. The majority of the participants, about (90.6%) have a religious belief, and about (5.6%) do not have any religious belief, while only about (3.6%) are do not know/not sure about religious beliefs. In the employment context, the majority of the participants about (34.7%) are employed full time (including self-employed or homemaker), about (31.6%) are students, about (12.6%) are retired, about (8.6%) are unemployed, and about (8.2%) are unable to work, while only about (4.3%) are doing a part-time job.

**TABLE 1:** Demographic characteristics of the survey participants (N-2200).

**Variables**	**Categories**	**Frequency**
Gender	Male	1086 (49.4%)
	Female	1096 (49.8%)
	Others	5 (0.2%)
	Prefer not to answer	13 (0.6%)
Age	18–24	830 (37.7%)
	25–34	193 (8.8%)
	35–44	390 (17.7%)
	45–54	414 (18.8%)
	55–64	136 (6.2%)
	65 years or older	237 (10.8%)
Education	Less than a high school degree	491 (22.3%)
	Associate degree	323 (14.7%)
	Bachelor’s degree	581 (26.4%)
	Graduate degree	595 (27.0%)
	Others	210 (9.5%)
Marital Status	Single (never married)	918 (41.7%)
	Married or in a domestic partnership	1106 (50.3%)
	Widowed	108 (4.9%)
	Divorced	58 (2.6%)
	Separated	10 (0.5%)
Religious Belief	Yes	1993 (90.6%)
	No	128 (5.6%)
	Do not know/Not sure	79 (3.6%)
Ethnicity	Han Ethnicity	2172 (98.8%)
	National minority	28 (1.2%)
Employment status before COVID-19	Employed full time (including self-employed or homemaker)	762 (34.7%)
	Employed part-time (including self-employed or homemaker)	95 (4.3%)
	Unemployed	188 (8.6%)
	Student	695 (31.6%)
	Retired	278 (12.6%)
	Unable to work	182 (8.2%)

### Multivariate Analysis

#### Measurement of the Conceptual Model

The reliability was examined of included factors and items in an online survey through Cronbach alpha values. Calculated values of Cronbach alpha were 0.945, 0.924, 0.990, 0.972, 0.955, and 0.917 (See Table 2). The Cronbach alpha’s standardized value for measuring the reliability is above 0.70, documented by Nunnally and Bernstein (1994). Thus, Cronbach alpha of each factor is considered reliable according to standard. Convergent and discriminant validity were determined through confirmatory factor analysis (CFA) for the proposed research model. Campbell and Fiske (1959) documented that the convergent technique assesses construct validity based on the multitrait-multimethod matrix (MTMM). The threshold level of factor loading values is 0.60, factor loading values of this work remained above than range, and the standard range of composite reliability (CR) values is above 0.70. The composite reliability (CR) values for each construct were calculated as 0.960, 0.943, 0.991, 0.975, 0.964, and 0.947 (see Table 2). The standard range of average variance extracted (AVE) is described as 0.50, and average variance extracted (AVE) values of this work are above than standard range of 0.858, 0.768, 0.791, 0.799, 0.817, 0.857 (Anonymous, 2018). The discriminant validity (DV) explained the measurement of constructs that theoretically did not relate one to another construct. Discriminant validation aims to provide any evidence of discrimination concerning dissimilarity of all factors (Campbell and Fiske, 1959). A useful evaluation of discriminant validity (DV) depends on a test of factors that are not positively correlated with other factors while calculating the overlap of measures on each other. Discriminant validity (DV) could be determined by comparing the Square Root of the average variance extracted (AVE) values of a factor with the correlation between constructs to other constructs. Square Root of average variance extracted (AVE) values should be higher than correlations (Campbell and Fiske, 1959). According to the discriminant validity (DV) analysis, all square root of average variance extracted (AVEs) is higher than correlations values (see Table 3), which shows good evaluation. Further, the multicollinearity of all items was assessed by variation influence factor (VIF) value. The acceptable range of variation influence factor (VIF) is less than 10, and <5 are considered acceptable (Henseler et al., 2014; Hair et al., 2017). For this work, variation influence factor (VIF) values are less than < 5, which means there is no multicollinearity problem among all formative constructs, as mentioned in the given Table 2.

**TABLE 2:** Display the measurement of research model with convergent validity (N-2200).

**Constructs**	**Items**	**Loadings**	**VIF**	**Cα**	**SCR**	**AVE**
Behavior and Attitude	BA1	0.923	3.89	0.945	0.960	0.858
	BA2	0.927	4.06			
	BA3	0.927	4.08			
	BA4	0.929	4.18			
COVID-19 Lock Down Policy	CLP1	0.893	3.21	0.924	0.943	0.768
	CLP2	0.884	2.92			
	CLP3	0.905	3.51			
	CLP4	0.819	2.16			
	CLP5	0.878	2.89			
Emotional Control	EC1	0.883	4.58	0.990	0.991	0.791
	EC10	0.889	4.65			
	EC11	0.884	4.55			
	EC12	0.890	4.68			
	EC13	0.894	4.90			
	EC14	0.888	4.59			
	EC15	0.886	4.41			
	EC16	0.886	4.58			
	EC17	0.888	4.68			
	EC18	0.887	4.65			
	EC19	0.892	4.96			
	EC2	0.886	4.54			
	EC20	0.891	4.82			
	EC21	0.893	4.82			
	EC22	0.894	4.92			
	EC23	0.895	5.05			
	EC24	0.885	4.56			
	EC25	0.898	5.07			
	EC26	0.892	4.87			
	EC27	0.895	4.92			
	EC28	0.894	4.90			
	EC3	0.883	4.49			
	EC4	0.888	4.61			
	EC5	0.882	4.52			
	EC6	0.890	4.73			
	EC7	0.886	4.67			
	EC8	0.885	4.57			
	EC9	0.892	4.97			
Lockdown Period Psycho-Impact	LPPI1	0.890	4.04	0.972	0.975	0.799
	LPPI10	0.899	4.36			
	LPPI2	0.891	4.14			
	LPPI3	0.896	4.31			
	LPPI4	0.893	4.07			
	LPPI5	0.895	4.15			
	LPPI6	0.891	4.02			
	LPPI7	0.898	4.38			
	LPPI8	0.896	4.30			
	LPPI9	0.886	3.89			
Physical Activity during Lockdown	PA1	0.911	4.15	0.955	0.964	0.817
	PA2	0.895	3.58			
	PA3	0.901	3.87			
	PA4	0.906	4.02			
	PA5	0.899	3.71			
	PA6	0.911	4.09			
Self-Belief	SB1	0.928	3.37	0.917	0.947	0.857
	SB2	0.923	3.09			
	SB3	0.927	3.30			

*BA = behavior and attitude, CLP = COVID-19 lockdown policy, EC = emotional control, LPPI = lockdown period psychological impact, PA = physical activity, and SB = self-belief.*

**TABLE 3:** Display discriminant validity analysis (N-2200).

	BA	CLP	EC	LPPI	PA	SB
BA	**0.926**					
CLP	–0.793	**0.876**				
EC	0.827	–0.769	**0.889**			
LPPI	–0.843	0.727	–0.803	**0.894**		
PA	0.743	–0.657	0.711	–0.678	**0.904**	
SB	0.669	–0.620	0.705	–0.667	0.535	**0.926**

*BA = behavior and attitude, CLP = COVID-19 lockdown policy, EC = emotional control, LPPI = lockdown period psychological impact, PA = physical activity, and SB = self-belief.*

#### Structural Equation Model

The model fitness of this work was examined by standardized-root-mean-square-residual (SRMR); Normed fit index (NFI), and chi-square (χ^2^) values. Standardized-root-mean-square-residual (SRMR) value is a standardized-residuals index that developed among observed covariance and hypothesized matrices, which shows model fitness (Brown, 2006; Chen, 2007). The acceptable range of standardized-root-mean-square-residual (SRMR) value is less or equal to 0.08. According to the results, the estimated standardized-root-mean-square-residual (SRMR) value is 0.076, acceptable as a good model fit. The normed fit index (NFI) value is 0.954, and chi-square (χ^2^) shows the value of 7425.338, as shown in Table 4. Standard beta was calculated to examine the significance level of the proposed hypothesizes. The beta value explains the possible variation of the dependent factor from the independent factor. According to the hypothesized research model, the standardized beta (β) value for each relationship was calculated (see Table 5). If beta (β) values are high and significant, then the substantial effects of endogenous latent variables will be considered high. Further, the T-statistics method was used to verify the significance of the beta value for each path. B Bootstrapping technique was used to obtained beta (β) value to assess and evaluate the significance level of proposed relationships.

**TABLE 4:** Model fit summary **(N-2200)**.

Statistical Tests	**Estimated Model**
SRMR	0.076
d_ULS	23.087
d_G	0.654
χ2	7425.338
NFI	0.954

*SRMR = Standardized-root-mean-square-residual, d_ULS = Unweighted least squares discrepancy, d_G = Geodesic discrepancy, χ2 = Chi-square, NFI = Normed fit index.*

**TABLE 5:** Final results for the standard beta, T-statistics, and *P*-values (N-2200).

**Hypothesis’s**	**Std. Beta (β)**	**T-Statistics**	***P*-Values**	**Decision**
CLP - > BA	–0.793	57.835	0.000	confirmed
CLP - > EC	–0.769	46.766	0.000	confirmed
CLP - > LPPI	0.727	43.968	0.000	confirmed
CLP - > PA	0.101	40.965	0.000	confirmed
CLP - > SB	–0.620	32.078	0.000	confirmed
BA - > PA	0.408	10.103	0.000	confirmed
EC - > PA	0.261	6.729	0.000	confirmed
SB - > PA	–0.033	1.202	0.230	Not-confirmed
LPPI - > PA	–0.073	1.731	0.084	Not-confirmed

*BA = behavior and attitude, CLP = COVID-19 lockdown policy, EC = emotional control, LPPI = lockdown period psychological impact, PA = physical activity, and SB = self-belief.*

**FIGURE 3:**
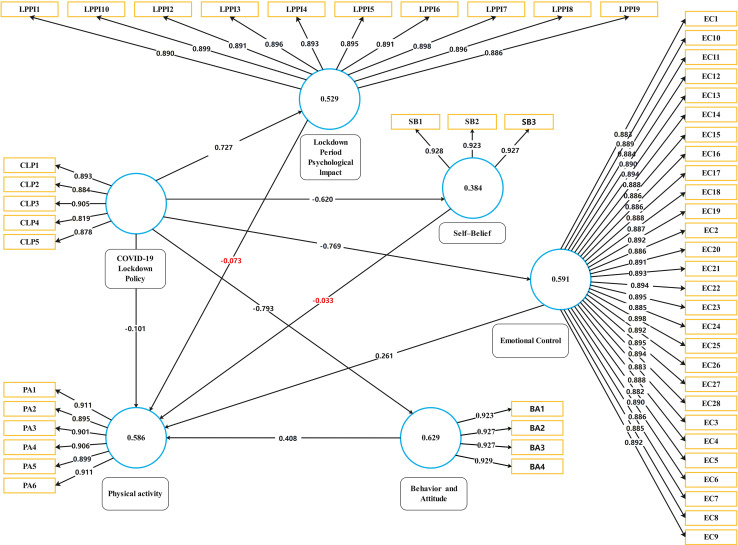
PLS-SEM results.

**FIGURE 4:**
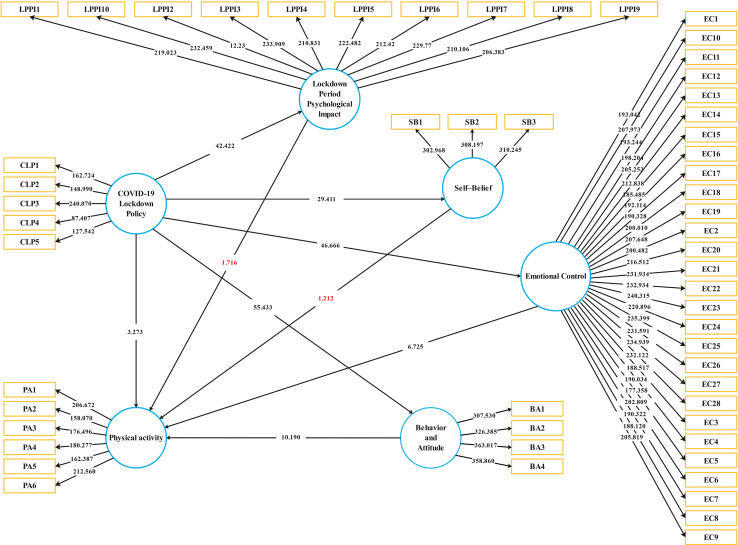
PLS-bootstrapping T-values and observed variables relationship of the study.

Table 5 and Figure 3 show all beta (β) values of the structural model’s proposed relationships for this work. Figure 4 shows the PLS-bootstrapping T-values and observed variables relationship. The results for H1 statistically document that the COVID-19 lockdown policy reveals a significant negative impact on a person’s behavior and attitude. According to results, H1 is supported with β = −0.793, *t* = 57.835, *p* = 0.000, which claimed a negative association betweenCOVID-19 lockdown policy (CLP) and behavior and attitude (BA). Results for H2 reveals that the COVID-19 lockdown policy have negative influence on emotional control with β = −0.769, *t* = 46.766, *p* = 0.000 and it is supported. H3 documented a significant positive relationship between COVID-19 lockdown policy (CLP) and lockdown period psychological impact (LPPI), which means lockdown policy, is the main reason to increase the lockdown psychological impact. COVID-19 lockdown policy (CLP) have negative influence on physical activity (PA) (H4) and self-belief (SB) (H5) with β = 0.101, *t* = 32.766, *p* = 0.000 and β = −0.620, *t* = −6.766, *p* = 0.000 respectively. H6 stated that there is a positive impact of behavior and attitude toward physical activity. The results for H6, behavior and attitude (BA) affecting the physical activity (PA) with β = 0.401, *t* = 10, *p* = 0.000 which is supported.

## Discussion

### Psychological Impact of COVID-19 Home Confinement

The physical environment has a significant impact on behavior and attitude. A specific kind of environment, such as home confinement during the lockdown, created a sudden terror, which negatively impacted human nature. In several countries across the world, the COVID-19 coronavirus pandemic has altered ways of life. Therefore on January 26, 2020, the World Health Organization (WHO) announced a greater risk of an outbreak in China and other regions (Fan et al., 2020). According to the study results, (β = −0.793, *t* = 57.835, *p* = 0.000) claimed that there is a negative association between COVID-19 lockdown policy (CLP) and behavior and attitude (BA). Through an online survey of 4,872 Chinese people, a rapid assessment was carried out. The results showed a high prevalence of mental health problems among the general population, particularly anxiety and depression, negatively impacting behavior and attitude (Gao et al., 2020). A significant association has been reported by Li et al. (2020) between COVID-19 and different psychological aspects of human nature. In this context, Extreme long-term stress can trigger neuromuscular and autoimmune malfunction, which would impact and undermine the human’s natural functions, leading to decreased throughout the body’s capacity to prevent infection (Miller and Cohen, 2001). The study results show that there is a significant positive relationship (β = −0.657, *t* = 32.766, *p* = 0.000 and β = −0.620, *t* = −6.766, *p* = 0.000) among COVID-19 lockdown policy (CLP) and lockdown period psychological impact (LPPI) which means lockdown policy is the main reason to increase the lockdown psychological impact on Wuhan residents. In line with the results, as mentioned earlier, a higher prevalence of depression among young people has also been reported in recent studies (Huang and Zhao, 2020). Mental health issues among the general population in China were also triggered by the outbreak of COVID-19 (Bao et al., 2020). Further, the study results show that there is a negative influence of COVID-19 lockdown on emotional control (β = −0.769, *t* = 46.766, *p* = 0.000). Pérez-Fuentes et al. (2020) reported that the perceived threat from COVID-19 positively impacted moods of frustration-depression, anxiety, and hatred-hostility, while panic and resentment-hostility had a significant positive impact on the perception of the epidemic hazard. Time spent under quarantine measures has had a detrimental effect on emotions, and this is consistent with previously reported work on the mental health consequences of the COVID-19 lockdown (Ozamiz-Etxebarria et al., 2020). The impact of such isolation on mental health and wellbeing at personal and population levels is several times greater than physical suffering. Public lockdown enforced by national lockdown systems can generate mass panic, anxiety, and distress due to factors such as cornering feelings and loss of power (Brooks et al., 2020).

### Physical Activity Participation During COVID-19 Home Confinement

Some researchers have shown with the COVID-19 epidemic that there is a good health argument for maintaining physical exercise in the home to preserve a balanced immune system function in the current unstable environment. Sports and physical activity contribute to health wellbeing. As lockdown imposed in Wuhan, public places such as dancing squares, playgrounds, and gymnasium were closed to contain the transmission of COVID-19. With the WHO’s consultation, the Chinese Government announced various Chinese citizens’ measures to keep physically fit while staying at home. Physical activity is one of the preventive measures which the Chinese Government announced. For certain disorders, including cardiovascular diseases and metabolic syndrome, physically active people are less at risk (Hamilton et al., 2014). The study results show a positive impact of behavior and attitude toward physical activity (β = 0.401, *t* = 10, *p* = 0.000). Moreover, COVID-19 lockdown policy (CLP) have negative influence on physical activity (PA) (H4) and self-belief (SB) (H5) with (β = −0.657, *t* = 32.766, *p* = 0.000) and (β = −0.620, *t* = −6.766, *p* = 0.000) respectively. Restrictions on physical movement also impact physical activity, and further social isolation also harms mental health wellbeing (Wilke et al., 20209). Social alienation is correlated with greater chronic illness morbidity and higher mortality from all causes. More than 30% of this impact can be caused by unhealthy health habits such as smoking and decreased physical activity (Valtorta et al., 2016).Moreover, COVID-19 lockdown policy (CLP) have negative influence on physical activity (PA) (H4) and self-belief (SB) (H5) with (β = −0.657, *t* = 32.766, *p* = 0.000). The negative influence of the COVID-19 lockdown policy (CLP) on physical activity (PA) shows a decline in daily physical activity. COVID-19 declared a global pandemic, and lockdown imposed across the world in different countries show the decline of physical activity. A study conducted in Canada and indicates a significant decline in step walking, moderate-to-vigorous physical activity (MPVA), and light physical activity (LPA) (Di Sebastiano et al., 2020). In the same paradigm, a study reported that overall physical activity and outdoor physical activity declined due to COVID-19 lockdown (Lesser and Nienhuis, 2020). The decreased levels of physical activity (PA) and sedentary lifestyle activity have been recognized as major problems arising from the outbreak while home detention (Bentlage et al., 2020). In the context of the above results, similar findings have been reported in a study regarding physical activity during Spain’s lockdown period. According to the study results, the Spanish population’s physical activity level declined in the first week of lockdown from 60.6% to 48.9% (López-Bueno et al., 2020). Health consciousness behavior and attitude play a significant role in the formation of a healthy lifestyle. A study conducted in China that explores the relationship between health consciousness and home-based exercise during the COVID-19 pandemic reported similar results as mentioned above (Pu et al., 2020). A significant driver of home-based exercise is health consciousness. The study participant’s attitude and behavior are found positive toward physical activity due to health consciousness.

## Conclusion

The COVID-19 pandemic created a panic across the globe when a severe lockdown was implemented in Wuhan. COVID-19 home confinement during the lockdown period made negative psychological impacts that further affect the socio-emotional state of the people. Health-based fears due to COVID-19 resulted in the shape of poor health wellbeing. Our study results show that due to the psychological impacts of COVID-19, people faced anxiety, depression, and an imbalance of emotional control state. Physical activity is a natural nutrient to cope with psychological issues and improve the human immune system’s efficiency. Participation in physical activity is also affected due to the lockdown policy. According to the study results, the COVID-19 lockdown policy negatively influences physical activity and shows a decline in daily physical activity. Physical movement restrictions harm physical activity, especially on outdoor physical activity. Physical inactivity increased due to lockdown, which was further associated with psychological and health wellbeing issues. Overall regular physical activity has preventive wellbeing effects, especially in health-related psychological problems. Consequently, alternatives should also be provided whenever possible to be physically involved in the outdoors during public health restrictions.

### Study Limitations

To participate in the survey, the respondent must be 18 years old and be a literate person. The convenience sampling technique was used under the non-probability sampling (NPS) method according to the study’s objective and nature. The study’s results can’t be generalized to the whole population because it’s hard to replicate the convenience sample results.

## Data Availability Statement

The datasets for this manuscript are not publicly available. Requests to access the datasets should be directed to the corresponding author.

## Ethics Statement

The studies involving human participants were reviewed and approved by Ethics Committee of Soochow University, Suzhou, China. The patients/participants provided their written informed consent to participate in this study.

## Author Contributions

RM developed the study model and hypothesis and drafted the manuscript. XS and YW collected the data through conducting a survey. ZAS and SM analyzed and interpreted the data. WI and BS drew the figures. SK did the proofreading.

## Conflict of Interest

The authors declare that the research was conducted in the absence of any commercial or financial relationships that could be construed as a potential conflict of interest.
